# Assessing the Occurrence of Waterborne Viruses in Reuse Systems: Analytical Limits and Needs

**DOI:** 10.3390/pathogens8030107

**Published:** 2019-07-22

**Authors:** Charles P. Gerba, Walter Q. Betancourt

**Affiliations:** Water and Energy Sustainable Technology (WEST) Center, The University of Arizona, 2959 W. Calle Agua Nueva, Tucson, AZ 85745, USA

**Keywords:** virus, infectivity, cell culture, molecular methods, wastewater, reuse

## Abstract

Detection of waterborne enteric viruses is an essential tool in assessing the risk of waterborne transmission. Cell culture is considered a gold standard for detection of these viruses. However, it is important to recognize the uncertainty and limitations of enteric virus detection in cell culture. Cell culture cannot support replication of all virus types and strains, and numerous factors control the efficacy of specific virus detection assays, including chemical additives, cell culture passage number, and sequential passage of a sample in cell culture. These factors can result in a 2- to 100-fold underestimation of virus infectivity. Molecular methods reduce the time for detection of viruses and are useful for detection of those that do not produce cytopathogenic effects. The usefulness of polymerase chain reaction (PCR) to access virus infectivity has been demonstrated for only a limited number of enteric viruses and is limited by an understanding of the mechanism of virus inactivation. All of these issues are important to consider when assessing waterborne infectious viruses and expected goals on virus reductions needed for recycled water. The use of safety factors to account for this may be useful to ensure that the risks in drinking water and recycled water for potable reuse are minimized.

## 1. Introduction

Quantifying the number of infectious viruses in water and wastewater is necessary to determine the risks associated with exposure (e.g., ingestion) and in determining the degree of treatment needed to reduce these risks to an acceptable level [1,2,3]. For example, the state of California requires a 12-log_10_ reduction of all human enteric viruses in recycled waters for potable reuse applications [4]. To achieve this goal, knowledge of the number of infectious viruses in wastewater before treatment is needed. Infectious viruses are defined as those capable of replicating in cell culture and thus, have the potential to replicate in humans and animals and cause disease. In this review, the term infectivity is used in reference to the ability of methods to measure infectious viruses. This requires methods that can determine the number of infectious viruses. The purpose of this review is to provide an understanding of the limitations of current methods for assessing the infectivity of waterborne enteric viruses. We believe that this is essential for interpreting the data on viruses in water for persons involved in assessing needed technology for the treatment of recycled water for reuse applications while considering the associated risks. 

Before the development and application of molecular methods for the assessment of virus occurrence in water, animal cell culture was the only practical method available. Virus growth in cell culture indicates the potential for the virus to replicate in humans and cause disease. Enteroviruses were found to readily grow in cell culture from the earliest days of techniques for maintaining animal cells in the laboratory. Because they were so easily cultivated, most of our historic knowledge on enteric virus behavior in water and removal by water/wastewater treatment processes is based on enteroviruses. The safety associated with vaccine poliovirus strains allowed for bench- and pilot-scale testing of treatment processes under controlled conditions. However, enteroviruses have rarely been associated with waterborne disease, and today, we know they are only a small fraction of the viral community found in wastewater that is capable of causing illness in humans [3,5]. This has been in part revealed through the application of the quantitative polymerase chain reaction (qPCR) assay and more recently by viral sewage metagenomics [6,7,8,9]. Unfortunately, these methods cannot directly detect the infectivity of waterborne viruses. Various approaches have been developed to assess infectivity of waterborne enteric viruses using molecular methods, but they are specific to the virus and the mechanism of virus inactivation [10,11,12]. The mechanism of virus inactivation may vary by the type of virus, disinfectant, and other methods which may make the virus incapable of replication [13,14]. Thus, there is no universal method which can substitute for cell culture assessing viral infectivity in humans and animals. 

## 2. Factors Affecting Virus Infectivity in Cell Culture

### 2.1. Type of Cell Culture (Continuous vs. Primary)

Two types of cell culture have been used for the detection of viruses in water. Primary cell cultures originate directly from the organs of animals and humans. The most commonly used cell cultures in virology derive from primates, including humans and monkeys; rodents, such as hamsters, rats, and mice; and birds, most notably chickens [15,16]. Moreover, cells from a primary culture may be subcultured to obtain a large number of cells. Cultures established in this fashion from primary cell cultures are called secondary cultures [16]. They can only be passaged for a limited number of cell generations (usually 20 to 100) after which the cells cease to divide, then degenerate and die, a phenomenon called crisis or senescence. On the other hand, continuous cell lines may be passaged indefinitely as they originate from transformed cells that are no longer subject to senescence. Continuous cell lines are relatively easy to maintain because they can be passaged indefinitely and are the cell line of choice today for environmental virology research. Primary cells from human and nonhuman primates are the most sensitive to the widest variety of viruses which infect humans since these cells maintain many of the important markers and functions seen in vivo [17,18]. However, primary cells are not in common use today. Continuous cell lines from human and nonhuman primates are usually more restrictive to the types of viruses they can propagate (Table 1). This is because the cell surface must have specific receptors for the attachment and replication of the virus. The continuous cell line, Buffalo green monkey (BGM), was selected for use by the United States Environmental Protection Agency because certain coxsackieviruses (CV) and polioviruses (PV) grew well in this cell line, producing cytopathogenic effects with similar sensitivity to virus growth in primary cells [19]. BGM cells were found to be the most sensitive continuous cell line for the detection of enteroviruses [20] and have become the most commonly used cell line for the detection of enteric viruses in water and wastewater in the United States for over 30 years. While laboratory strains of echovirus will grow in this cell line, its use with environmental samples tends to favor the isolation of group B coxsackieviruses [20,21]. This may be due to the more rapid growth of group B coxsackieviruses in BGM cells [20]. An exhaustive comparison of cell lines and enteric virus susceptibility (16 cell lines against 105 different virus types) demonstrated a great deal of variability in cell susceptibility to virus type [22]. They found that not a single cell line could detect all enteroviruses, even of the same genus. In addition, with the control of poliovirus infections in the developed world and the elimination of the oral live poliovirus vaccine, vaccine strains of poliovirus are now absent in wastewater in most developed countries. Because live attenuated viruses replicate in the gut of vaccine recipients and spread person to person within a community, poliovirus was a common isolate in wastewater and sewage-polluted waters when vaccination was common from the mid-1950s until the mid-1990s in the United States. This is not surprising as vaccine poliovirus strains were selected for their ability to grow in high titers in cell culture.

The number of times a cell line has been passaged in the laboratory may also affect the ability of the virus to replicate (Table 2). Certain variants of the cells may be selected for over time because of their more rapid growth, which may be less or non-permissive to the replication of the virus. 

Over time cell cultures become less efficient for replication of certain types of viruses [26]. Previous studies reported that BGM cells became less efficient to coxsackievirus B3 (CVB3) and CVB4 but were still sensitive to poliovirus 1 [27]. This questions the use of positive virus controls in environmental assays. Use of any specific strain of a laboratory grown virus does not mean the cell line has not lost its ability to replicate viruses of the same group or naturally occurring viruses of the same type.

In the case of the double-stranded DNA adenovirus, it has been found that replication of the virus after ultraviolet light exposure is dependent upon the ability of the cell line to repair damage to the DNA [31]. UV light causes crosslinking of the DNA and can be repaired by enzymes in the host cell. This ability depends on the cell line, with some being more effective than others [31]. 

### 2.2. Cytotoxicity in Cell Lines

Virus concentrates from different water matrices (e.g., surface water, sewage, secondary or tertiary treated wastewater, groundwater) can contain compounds toxic to cell cultures used for the detection of infectious viruses. The cytotoxicity may be associated with metals, complex mixtures of compounds associated with microalgae or plants as well as the reagents used for virus concentration and recovery [32,33,34]. Numerous methods have been applied for reducing cytotoxicity associated with the produced concentrates including sample dilution, washing cell monolayers with saline solution after inoculation, freon extraction, and cationic polyelectrolyte precipitation or high-speed centrifugation followed by filtration of the samples through positively charged depth filters [35,36,37]. Studies have also revealed that sample concentrates toxic to cells may not be necessarily inhibitory to the RT-PCR analysis [38]. 

### 2.3. Virus Type

Viral growth in cell culture is limited by the ability of virions to attach to specific receptors on the surface of animal cells and their ability to replicate within the cells. For the enteroviruses and many of the enteric viruses, this results in morphological changes induced in individual cells or groups of cells by virus infection that can be easily recognized by light microscopy and collectively called cytopathic or cytopathogenic effect (CPE). However, there are viruses whose replication may be limited to one or a few adjacent cells with no obvious cytopathogenic effects [15]. Alternative approaches, such as immunofluorescence, immunoperoxidase, electron microscopy or polymerase chain reaction (PCR) assays, have been used for detection of viruses that produce CPE slowly or not at all in cultured cells [15,28]. How this limited growth can be equated to the risk of infection and illness in humans is uncertain. Continuous cell lines are not necessarily reflective of the cells within the human host and the ability of the virus to destroy cells or establish themselves as subclinical or latent infections. For example, coxsackieviruses may establish lifelong latent infections in humans [39]. 

Cell culture is also less permissive for the growth of naturally occurring viruses than laboratory-grown viruses. Viruses grown in the laboratory have been selected for their rapid growth in cell culture and the number of virions observed under an electron microscope versus number observed by CPE or plaque-forming units (PFU) is usually 1:2 to 1:100 depending on the virus and method of assay [40,41]. In the case of naturally occurring viruses in stool samples, this ratio may be as great as 1:46,000 [42,43]. The particle-to-PFU ratio of poliovirus ranges from 30 to 1000, which is similar for other members of the *Picornaviridae* family [42]. Passage of naturally occurring viruses in cultured cells usually results in the significant lowering of this ratio [43] as mutants, which replicate in the specific cell line selected. Comparing the ratio of viral particles to genomes detected by molecular methods has also been attempted. However, several limitations exist, e.g., not all virus types grow in one type of cell culture, and there are differences in the quantitative precision of the methods for estimation of virus particles and viral genomes. Previous studies attempted to determine the ratio of enteroviruses detected by reverse transcription-PCR versus the number of infectious viruses determined in the cell culture [44]. The ratio of virus genomes to infectious virions reported in the study was 1:200. This ratio is likely significantly underestimated because the cells were only observed for 5 days for CPE, and not all enteroviruses can grow in this cell line. Another study comparing integrated cell culture-PCR (ICC-PCR) and real-time quantitative PCR (qPCR) in sewage polluted waters found that greater numbers of adenoviruses were detected by ICC-PCR [45]. Using the improved cell line (293 CMV) for detecting enteric human adenoviruses (HAdVs), the replication of HAdV in the cell line was determined by measuring the production of viral mRNA and determining the levels of viral DNA [46]. The results of the study demonstrated the effectiveness of the new transactivated 293 CMV cell line for improved propagation and detection of HAdVs from environmental samples. The ratio of infectious adenovirus with the improved cell line varied from 1:13.7 to 1:22 [46]. In a similar study, it was found that the ratio of infectious adenovirus by cell culture infectivity determined by the detection of viral mRNA production varied from 1:11 to 1:381 in untreated sewage [47].

The degree of viral aggregation may also influence the underestimation of infective viruses in a sample. Aggregated viruses in cell culture are often only counted as one infectious virus as a result of only one countable plaque [48]. However, they may represent thousands of potentially infectious viruses and have a greater probability of infection when ingested [49].

Another complicating factor is that one group of viruses may grow faster than another or interfere with the replication of another group of viruses [50], which again underestimates the true number of infectious viruses able to replicate in one specific cell line.

## 3. Impact of Assay Methods on Virus Detection

The three most common methods for quantitative detection of virus replication in cell culture are the total culturable virus quantal assay (TCVQA) which requires computation of a most probable number (MPN), the plaque assay which quantifies the number of plaque-forming units in a virus sample as plaque-forming units (PFU), and the 50% tissue culture infective dose (TCID_50_) assay that quantifies the amount of virus required to produce CPE in, or kill 50% of virus-inoculated cultured cells in a multi-welled plate [16,51]. The TCVQA has been used for detection of enteric viruses in wastewater, but not all viruses will plaque or may require mixed cell types or pretreatment of cells before inoculation to form plaques [24]. Other limitations of the method include the difficulty to keep the monolayers beyond 5 to 7 days under an agar overlay, inability to perform a second passage, and laboratory strains which produce CPE in cell culture may not form plaques [21]. Numerous methods have been developed to determine the replication of viruses within cell culture (Table 3). None of these methods can detect all of the infective viruses in an environmental sample, even if the cell line is susceptible to the virus. As a first step, the virus must come into contact with a receptor on the cell membrane. Thus, the size of the inoculum (e.g., volume) of the sample, as well as a means to enhance contact with the cell membrane, are important steps in the efficiency of the assay for detecting infectious viruses. A previous study found that the optimal inoculum volume for poliovirus type 1 was one mL per 25 cm^2^ of cell monolayer [28]. A marked decrease in the number of plaques was observed when over 1 mL of sample was inoculated on this surface area. The numbers of infectious viruses can also be increased by using roller bottles [52]. Secondary passage on fresh cells, use of suspended cell culture, rotating or shaking the liquid in the cell culture flasks during incubation [53], and use of suspended-cell may increase the number of viruses detected. All of these methods increase the probability of contact of the virus with receptors on the cell surface, i.e., the suspended virus must come into contact with cells. However, the increase in titer or probability of isolation may be virus and type dependent. For example, the suspended cell culture technique was found to increase the titer of poliovirus type 1 almost 10 fold but had no significant effect on echovirus 1 titer in BGM cells [15]. The appearance of CPE also varies greatly with naturally occurring viruses taking longer than laboratory-grown viruses. This is because laboratory-grown viruses have been selected for rapid growth in cell culture, as previously discussed. While CPE for vaccine strain of poliovirus may take only 48 hours, natural isolates of other enteroviruses may take five days or longer. A previous study demonstrated that going from a two-week incubation to three weeks resulted in a 100-fold increase titer in adenovirus 2 [30]. In the case of adenovirus 2 exposed to UV light, the increase in titer was 140-fold. This suggests that the longer incubation period allows for greater time for the cell enzymes to repair UV light damage of adenovirus. The most common methods to assess viral infectivity are shown in Table 3. All of these involve the use of cell culture except PCR. The use of the plaque-forming unit method previously mentioned, which involves an agar overlay of the cell monolayer to reduce virus spreading, results in a more precise quantification of viruses able to form plaques. This is true of naturally occurring viruses which can require a second passage or even a third passage before the production of CPE. 

A variety of additives (Table 4) have been used to enhance viral infectivity in cell culture and to increase the range of susceptibility to a greater range of viral types [22]. For example, use of 5-ido-2’-deoxyuridine will result in plaque formation of adenovirus 1 and echoviruses [24]. Incorporation of enzymes is also known to enhance the infectivity of reoviruses in cell culture [40]. A secondary passage of environmental samples is often necessary for observation of CPE for some viruses [28]. This is because of the slower growth of naturally occurring viruses in cell lines and that not all the viruses in the sample will come into contact with the monolayer. Additional studies indicated that removal of the inoculum of poliovirus 1 from a cell flask containing a monolayer onto a fresh monolayer resulted in a 10-fold increase in titer of the virus (2300 to 24,000 most probable number) [41]. Passage a third time resulted in an additional increase in titer. For example, we have never observed the production of reovirus CPE in BGM cells in wastewater samples until a second or third passage [54]. Techniques using antibodies and PCR have the advantage of detecting virus replication without the production of CPE. Specific antibodies or primers are required for virus detection. PCR has the advantage in that replication of groups of viruses (i.e., enteroviruses, adenoviruses, rotavirus, etc.) can be identified, even if limited replication has taken place. Pool sera from multiple individuals has been used to determine the replication of viruses by an immunoperoxidase method, which increased the scope of viruses replicating in cell culture [28].

## 4. Molecular Methods for Assessing Virus Infectivity

### 4.1. Integrated Cell Culture-Polymerase Chain Reaction

Various molecular methods have been developed to more rapidly determine the growth of viruses in cell culture and ultimately for the detection of viruses, which may not produce CPE or exhibit limited growth [55,57,58,59]. This still requires that each inoculated cell culture flask be tested and that primers for each group of virus to be tested are available. The major advantage of this method is that replicating viruses can be detected in less time than observation of CPE or plaques and they can detect viruses which do not produce CPE. Generally, virus replication can be detected in 2 to 5 days after inoculation but depends on the virus type [60]. In the United States, a study found that the use of ICC-PCR resulted in an increase in positive samples of surface water from 17.2% (5/29) by CPE to 93.1% (27/29) [61]. Studies conducted in South Korea [62] also reported greater isolation of naturally occurring enteric viruses by ICC-PCR and detection of enteric viruses in treated tap water that was previously negative by CPE. Similarly, a study conducted in New Zealand [45] reported greater numbers of viruses detected in surface waters using ICC-PCR than by qPCR.

Assays targeting viral messenger RNA for detection of human adenoviruses in environmental samples have been developed [47,63] but have not been widely applied in ambient waters. In addition, a molecular beacon-based real-time PCR assay has been applied to identify intact enteroviral particles combined with a reporter cell system to determine viral replication. The reporter assay depended upon fluorescence emitted by single-stranded dual-label antisense oligonucleotide probes (i.e., molecular bacons) upon binding to the specified target (e.g., mRNA) [64,65].

### 4.2. Direct Molecular Methods for Detecting Virus Infectivity

Various methods have been developed to determine the potential infectivity of enteric viruses directly by molecular methods. The potential application of these methods and their limitations have been reviewed [59,66,67]. The success of such methods depends on knowledge of the mechanism of inactivation of a particular virus and the site of action of a particular disinfectant [2,68]. Different virus types and strains may have different sites of action for a particular disinfectant. Thus, one method that may work for RNA viruses may not work for dsDNA viruses. In addition, complicating this approach is that some viruses, such as adenoviruses, rendered non-infectious by ultraviolet light can use host cell enzymes to repair DNA damages on their genome [31,68]. Inactivated viruses can still cause infection in cells through multiplicity reactivation [69]. This occurs when two viruses with their nucleic acids damaged in different regions of their genomes infect the same host cell resulting in a complete genome capable of replication.

Intercalating dyes, such as propidium monoazide (PMA) and ethidium monoazide (EMA) in conjunction with qPCR (PMA-RT-qPCR and PMA-qPCR for RNA or DNA viruses, respectively), have been used to determine the potential infectivity of enteric viruses in water [11,70,71]. Treatment of virus suspensions with platinum (IV) chloride (PtCl4) has also been applied to discriminate between potentially infectious and thermally inactivated enteric hepatitis viruses in environmental samples [12,72,73]. Two hypotheses underlay the use of intercalating dyes (i) a virus with a damaged capsid is not infectious, (ii) intercalating dyes can reach and bind the genomes to block specifically the amplification of defective particles [68]. However, the success of these methods depends on knowledge of the mechanism of inactivation of a particular virus and the site of action of a particular disinfectant [2,68,74]. 

Another qPCR-based framework has been described and used to estimate virus infectivity [75]. The framework quantifies damage to the entire genome based on the qPCR amplification of smaller sections, assuming single-hit inactivation and a Poisson distribution of damage. The framework offers the potential to monitor the infectivity of viruses that remain nonculturable or not easily grown in cell culture, such as norovirus. 

## 5. Conclusions

Determining the concentration of infectious enteric viruses in water reuse systems will likely be problematic into the near future. No one cell culture system can detect all of the infectious viruses that may be present in an environmental sample. However, advances in molecular biology which allow us to detect the genome of viruses known to infect humans and animals in environmental samples have revealed that the number of viruses may be 100 to 1000 greater than that detected by cell culture [3,76]. This requires us to reassess what proportion of these viruses that are potentially infectious so that we can adequately assess the risk and design treatment systems to reduce the risk of exposure. The ratio of virus genome detected versus those detected by viral culture will be greatly affected by wastewater and wastewater treatment processes and will not be a constant value. For example, different disinfectants will affect different virus types differently (e.g., different sites of action on the viral capsid or genome), and the presence of resistant mutants or viruses capable of the use of host cell enzymes for repair (infectivity can be affected by choice of cell line). Perhaps the best approach at present is to use molecular methods to assess the presence of enteric viruses in untreated wastewater where most viruses can be expected to be infectious. This has been the approach for treatment requirements in water reuse applications for potable and non-potable purposes, including irrigation of crops traditionally consumed raw [4,77].

Another approach to consider is the use of a safety factor when estimating the true concentration of an infection virus in an environmental sample. This might be useful since no one method can detect all of the likely infectious virus present in environmental samples. When estimating risk from chemicals, it is common to take into consideration the uncertainty of using data on toxicity developed in animals to humans and the lack of data. Usually, safety factors of 10 to 100 are used to estimate acceptable levels of risk. While this may be useful for estimating levels of infectious virus in raw wastewaters, it becomes more problematic when dealing with treated wastewater and environmental waters. However, considering the factors outlined in this review affecting assays for enteric viruses that a safety factor of 10 would not be unreasonable. 

## Figures and Tables

**Table 1:** Susceptibilities of cell culture lines most commonly used for isolation and detection of waterborne enteric viruses.

Cell Line	ADENO	CV-A	CV-B	ECHO	PV	REO	ROTAV	ASTROV
Human Embryonic Kidney	++	+	+		+	+		
A549	++++							
Buffalo Green Monkey (BGM)	+	+	++++	++	+++	+++		
Human rhabdomyosarcoma	-	++	-	++	++			
Caco-2 *	+	+	++	?	+	+	+	+
								
PLC/PRC/5 **	++		++	++?				
								
HEL-299 ***	++	++	++	-	+			
RD	+	++	-	++	+	+		

Note: The number of + signs indicate the relative degree of replication of the virus in the specific cell line. A “–“sign indicates no replication. *[23] **[24,25] ***[26] ADENO: Adenovirus; CV-A: Coxsackievirus A; CV-B: Coxsackievirus B; ECHO: Echovirus; PV: Poliovirus; REO: Reovirus, ROTAV: Rotavirus; ASTROV: Astrovirus, RD: Rhabdomyosarcoma titers in cell culture. Thus, it should be recognized that much of our information on viruses in water and the effectiveness of treatment processes comes from a very limited group of enteroviruses. The question mark indicates potential replication of the virus in the corresponding cell line.

**Table 2:** Factors that influence the infectivity of viruses in cell culture.

Factor	Remarks	References
Type of virus	Not all viruses can be grown in cell culture	[26]
Type of cell line	Not all viruses can be grown in the same cell culture	[26]
Number of times cell line has been passed in the laboratory	Cells may lose their sensitivity to virus infectivity after prolong passage in the laboratory; this may be virus-specific	[26,27]
Laboratory grown versus naturally occurring viruses	Laboratory grown viruses have been adapted for rapid growth and infectivity in cell culture.	[28]
Effectiveness of host cell repair enzymes	Host cell repair enzymes can repair damage to double-stranded DNA viruses after exposure to UV light. This may vary with cell line	[29]
Observation time for production of CPE	This may take days to weeks	[30]

**Table 3:** Most common methods used to assess viral infectivity in cell culture from environmental samples.

Method	Reference
Plaque form unit (PFU)	[28]
Most probable number by cytopathogenic effects (MPN)	[28]
Tissue culture infectious dose 50% (TCID_50_)	[28]
Integrated Cell Culture polymerase chain reaction (ICC-PCR)	[55]
Detection of messenger RNA	[47]

**Table 4:** Methods used to enhance cell culture infectivity to increase in virus numbers quantified.

Method	Virus	Increase in titer	Reference
5-iodo-2’-deoxyuridine	enteroviruses	Range of 0.7- to 3.3-fold increase in titer depending on virus type	[24]
Suspended cell culture agar method	Various enterovirus, wastewater and river water, and filtered water	Average 5.6-fold by plaque-forming unit method; range 0.1 to 23.3; 10 to 100 fold with BGM cells with polluted river water	[21,26]
Double agar overlay	Various enteroviruses and sewage isolates	7.7- to 12-fold over monolayer depending upon the virus	[56]
Rocking	Poliovirus 1	16% to 23% more plaques for rocked flasks. Increases rate of virus adsorption to cells	[53]
Adsorption time	Poliovirus 1	Maximum at 2 hours: ~2.5-fold from 30 min to 2 hrs for rocked flasks	[53]
Soluble proteins	Poliovirus 1	80% reduction of plaques in the presence of 3% beef extract compare to phosphate-buffered saline	[53]
Size of inoculum	Poliovirus 1	Inoculum of greater than one ml/25 cm^2^ resulted in decreasing numbers of plaques and MPN	[28,53]
Flask vs. roller bottlePlaques vs. CPE	Poliovirus 1	Greater sensitivity when detecting low levels of virus in a sample	[52]
Sequential passage	Poliovirus 1	Titer can increase by 10- to 100-fold	[41,52]
